# Spontaneous Deposition of Prussian Blue on Multi-Walled Carbon Nanotubes and the Application in an Amperometric Biosensor

**DOI:** 10.3390/nano2040428

**Published:** 2012-11-27

**Authors:** Yanli Yao, Xiaoyun Bai, Kwok-Keung Shiu

**Affiliations:** Department of Chemistry, Hong Kong Baptist University, Kowloon Tong, Hong Kong; Email: xli0207@sina.com (Y.Y.); 11466731@life.hkbu.edu.hk (X.B.)

**Keywords:** Prussian blue, spontaneous deposition, carbon nanotubes, glucose determination

## Abstract

A simple method has been developed for the spontaneous deposition of Prussian blue (PB) particles from a solution containing only ferricyanide ions onto conducting substrates such as indium tin oxide glass, glassy carbon disk and carbon nanotube (CNT) materials. Formation of PB deposits was confirmed by ultraviolet-visible absorption spectrometry and electrochemical techniques. The surface morphology of the PB particles deposited on the substrates was examined by atomic force microscopy and scanning electron microscopy. CNT/PB composite modified glassy carbon electrodes exhibited an electrocatalytic property for hydrogen peroxide reduction. These modified electrodes exhibited a high sensitivity for electrocatalytic reduction of hydrogen peroxide at −0.05 V (*vs.* Ag|AgCl), probably due to the synergistic effect of CNT with PB. Then, CNT/PB modified electrodes were further developed as amperometric glucose biosensors. These biosensors offered a linear response to glucose concentration from 0.1 to 0.9 mM with good selectivity, high sensitivity of 0.102 A M^−1^ cm^−2^ and short response time (within 2 s) at a negative operation potential of −0.05 V (*vs.* Ag|AgCl). The detection limit was estimated to be 0.01 mM at a signal-to-noise ratio of 3.

## 1. Introduction

Prussian blue (potassium iron(III) hexacyanoferrate(II)) and its analogues form an important class of insoluble mixed valence compounds [1,2,3]. Prussian blue (PB) has a three dimensional structure, consisting of Fe(III) and Fe(II) ions on cubic lattice sites. Iron(II) ions are surrounded octahedrically by the carbon atoms of cyanide ions, while iron(III) ions are linked at the nitrogen ends of the cyanides [4]. PB has received much attention for its electrochemical, photophysical, and magnetic properties, as well as the ease of preparation and low cost. As one of the promising electrochemically active inorganic compounds, PB can be applied to the development of electrochemical devices [5], fuel cells [6,7,8], electrochromic devices [9,10,11,12], magnetic materials [13,14,15] and in the bioelectrocatalytic fields [16,17,18]. Due to the comparably high catalytic activity and selectivity to biocatalysis, PB has been considered as an “artificial peroxidase” [13]. PB can be reduced to “Prussian white”, while oxidation of PB produces “Prussian yellow”. Prussian blue showed pronounced catalytic effect for the oxidation-reduction of low molecular weight molecules such as O_2_ and H_2_O_2_ [19]. The three dimensional structure of PB allowed the diffusion of H_2_O_2_ and O_2_ through the crystal lattice [19,20]. According to Itaya and coworkers [20], reduction of H_2_O_2_ was readily catalyzed by the divalent iron ion centers in the PB film. On the other hand, PB offered better activity and selectivity for H_2_O_2_ detection than at the Pt surface in neutral medium and in the presence of O_2_ [21]. The application of PB in the construction of chemical sensors and biosensors has been demonstrated [22,23,24,25].

PB can be deposited on an electrode surface by different electrochemical and chemical methods [1,2,26]. Neff [27] reported the deposition of PB film on a clean electrode surface such as Pt, Au, Al and graphite by incubating the electrodes in a solution containing ferric chloride and potassium ferricyanide. PB particles can be mechanically incorporated in carbon paste materials to exhibit analytical activity with low sensitivity [28]. Recently, Hu *et al.* [29] reported the preparation of PB by a photocatalytic method. Usually, PB deposits obtained by these methods show poor stability or low electrocatalytic activity. However, Moscone and coworkers [24] developed biosensors with long-term stability by incorporating a mixture of mineral oil or solid paraffin with carbon paste and PB particles in the electrodes.

Carbon nanotube (CNT) materials have attracted much attention since their discovery due to their unique mechanical, chemical, physical and electronic properties [18,30,31]. CNT materials are also capable of improving electron transfer rates for enzymes and other biomolecules. Many literature reports demonstrated that CNT modified electrodes offered electrocatalytic effects to biomolecules such as dopamine (DA) [32,33], β-nicotinamide adenine dinucleotide (NADH) [34,35], glucose [36,37] and H_2_O_2_ [38] based on the ability of CNT to improve electron transfer rates of enzymes and other biomolecules.

Recently, electrochemical biosensors based on the combination of CNT and PB on electrodes have gained great interest for the excellent synergistic electrocatalytic effect offered by the composite CNT/PB material [3,18,39,40]. Preparation of the CNT/PB composite usually employed direct reaction/interaction between ferric species and ferricyanide ions [2,19] through chemical or electrochemical methods, followed by mixing with or deposition on CNT materials. Only a few reports describe the deposition of PB on CNT from a solution containing ferricyanide ions alone. A simple method for the spontaneous deposition of PB on a CNT modified glassy carbon electrode in a solution containing only ferricyanide ions is described in this work. A glassy carbon electrode modified with the CNT/PB material (GC/CNT/PB) showed better electrocatalytic behavior for the reduction of H_2_O_2_ compared to electrodes incorporated with either PB or CNT alone, indicating a synergistic effect when both PB and CNT were present. The GC/CNT/PB electrode was applied to the development of an amperometric glucose biosensor by co-immobilization of glucose oxidase (GOD) and polyphenylenediamine (PPD) at the surface of the GC/CNT/PB modified electrode. The performance of the resulting GC/CNT/PB/PPD-GOD electrode for glucose detection has been evaluated.

## 2. Results and Discussion

### 2.1. Deposition of Prussian Blue on Different Electrode Substrates

Deposition of PB on different electrode substrates was investigated. The substrates examined included silica glass, ITO glass, CNT-modified ITO glass, and CNT-modified GC disk. The electrode substrates were incubated in 1 mM K_3_Fe(CN)_6_ in 0.1 M KCl (pH 1.6) overnight. Deposition of PB particles on the substrates was evidenced by color change from pale yellow to dark blue. A possible mechanism for this reaction is presented in Section 2.2.

**Figure 1:**
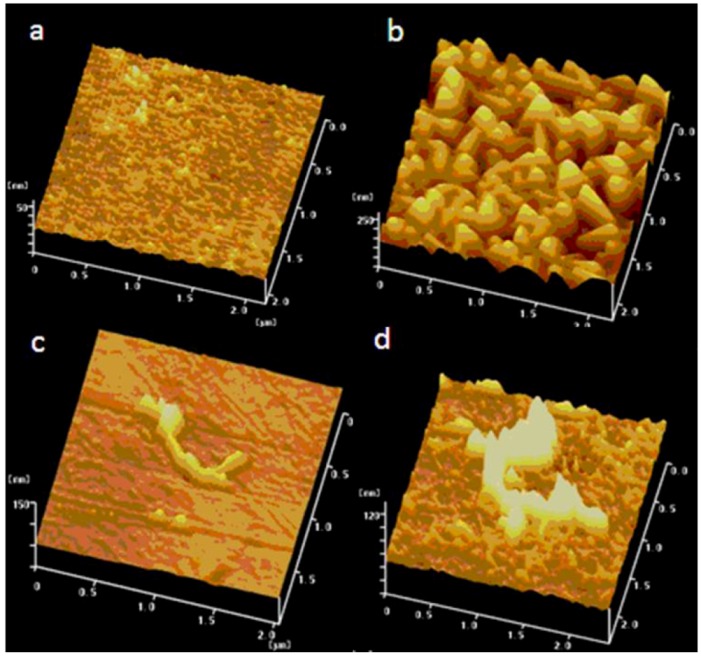
Atomic force microscopy (AFM) images of (**a**) bare ITO glass, (**b**) Prussian blue (PB) deposited on ITO surface, (**c**) ITO deposited with carbon nanotube (CNT) materials, and (**d**) PB deposited on ITO/CNT surface.

The effects of PB deposition on the surface of the substrates were investigated by atomic force microscopy (AFM). Figure 1 displays a series of AFM images of ITO glass slides modified by different materials. Small bright dots of ITO clusters were clearly observed on the bare ITO glass (Figure 1a). After incubating the same ITO glass in an acidic Fe(CN)_6_^3−^ solution for 24 h, a layer of PB clusters was observable on the ITO surface, as shown in Figure 1b. Figure 1c shows the AFM image of CNT deposits on the ITO glass. Similarly, after incubating the ITO/CNT in acidic Fe(CN)_6_^3−^ solution for 24 h, an increase in the tube diameter was apparent. It indicated that PB particles were attached onto the surface of CNT deposits, as shown in Figure 1d. On the other hand, when a non-conducting silica glass was incubated in acidic Fe(CN)_6_^3−^ solution for 24 h, no evidence of PB deposits was observed on this substrate. Experimental results suggested that PB would deposit spontaneously on the conducting substrates. However, when the non-conducting silica glass was placed in the acidic Fe(CN)_6_^3−^ solution for several days, a blue film was found on the glass surface, indicating the slow formation of PB film [29].

**Figure 2:**
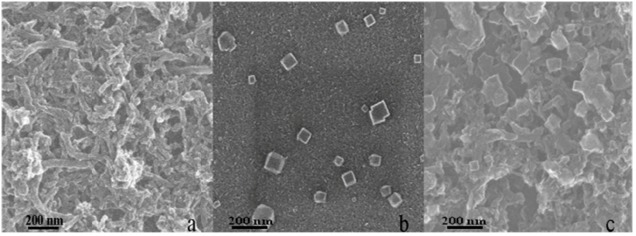
Scanning electron microscopy (SEM) images of different electrodes: (**a**) GC/CNT, (**b**) GC/PB, and (**c**) GC/CNT/PB.

The morphology of PB deposits on the GC/CNT and bare GC electrode surface were investigated by scanning electron microscopy (SEM), and the average size of the PB deposits was estimated. Figure 2a shows the SEM image of purified CNT materials dispersed on a GC surface. The cylindrical CNT materials exhibited a three-dimensional network structure with an average diameter of approximately 40 nm. PB deposits were also formed on the GC surface when a bare GC electrode was immersed in the acidic Fe(CN)_6_^3−^ solution overnight. SEM results demonstrated that spontaneous deposition of PB on different substrates from a single acidic Fe(CN)_6_^3−^ solution had been realized. Figure 2b displays a small number of PB particles formed on the GC surface. The PB particles exhibited a cubic crystal form with side length between 60 and 150 nm. When the GC/CNT electrode was immersed in acidic Fe(CN)_6_^3−^ solution for 24 h, PB particles were deposited on the GC surface, as evidenced by the SEM image shown in Figure 2c. This result was consistent with the deposition of PB on the ITO/CNT surface as shown by the AFM studies. PB particles were successfully immobilised on different substrate surfaces from a solution containing only Fe(CN)_6_^3−^ ions. A larger number of PB particles and clusters were homogeneously distributed throughout the GC/CNT surface with size between 70 and 200 nm, slightly larger than the PB particles formed on bare GC.

**Figure 3:**
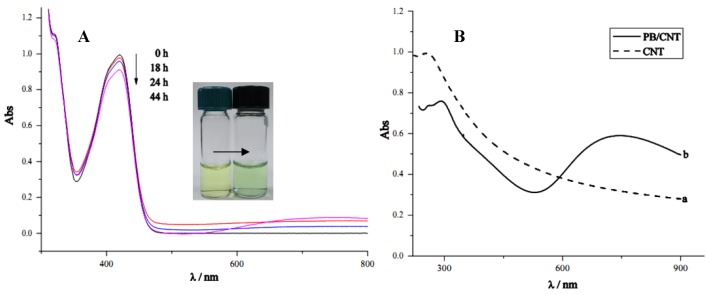
(**A**) Change in UV–Visible absorption spectra of ferricyanide solution after subjected to PB deposition for different periods of time; (**B**) UV–Visible absorption spectra of quartz slides with (**a**) CNT, and (**b**) PB deposited on CNT.

The formation of PB deposits was also supported by UV-Visible spectroscopy. Figure 3A shows the absorption spectra of the acidic Fe(CN)_6_^3−^ solution when subjected to spontaneous deposition of PB for different periods of time. The characteristic absorption peak of Fe(CN)_6_^3−^ at approximately 420 nm decreased in absorbance on increasing deposition time, and the solution color changed from yellow to green. After the Fe(CN)_6_^3−^ solution was subjected to PB deposition for 44 h, a new broad absorption band appeared at approximately 738 nm, corresponding to the characteristic absorption of PB materials [2], which indicated the formation of PB from the solution containing Fe(CN)_6_^3−^ only.

When a non-conductive silica glass was incubated in the acidic Fe(CN)_6_^3−^ solution for 24 h, no PB deposits were observed on this substrate. However, the formation of PB did occur after incubating the silica glass in the solution for several days, indicating a slow deposition of PB on non-conducting substrates. The result indicated possible ITO- and/or CNT-catalysed formation of PB. Purified CNT treated with strong acid can yield plenty of oxygen-containing groups (such as –OH, –COOH) [41,42]. The presence of the oxygen-containing functional groups is known to favor the nucleation of Fe(II) to form PB on a CNT surface [43].

Figure 3B shows the ultraviolet-visible absorption spectrum of the CNT materials deposited on quartz slides. The CNT-coated quartz slide was characterized by an absorption peak at approximately 255 nm, as shown in Figure 3Ba), consistent with reports in the literature [44,45]. For the CNT/PB composite material, the absorption peak at 734 nm resulted from the intense charge transfer absorption band of the mixed valence PB materials [46,47] shown in Figure 3B(b). Experimental results confirmed the presence of PB deposits on the CNT materials. Additionally, the absorption peak of CNT shifted to 292 nm, possibly due to the change in the interfacial electron density at the surface of CNT resulting from the interaction between oxygen-containing functionalities with Fe(II) ions.

### 2.2. Mechanism for the Spontaneous Deposition of Prussian Blue

The mechanism of PB deposition on a conducting substrate is under investigation. Metal species have been previously reported to undergo spontaneous reduction at carbon nanotube materials [48,49]. Formation of Prussian blue was catalytically promoted by reducing MWCNT. According to Zhang and coworkers [2], the successful deposition of PB particles on a substrate from a solution containing only ferricyanide species occurs mainly due to the dissociation of Fe(CN)_6_^3−^ to give Fe^3+^ species (Reaction 1 shown below), with the evolution of HCN gas as the driving force of the reaction.

Fe(CN)_6_^3^^−^ + 6H^+^ → Fe^3+^ + 6HCN (g) i001 (1)

Fe^3+^ + 1/2H_2_O → 1/4O_2_ (g) + H^+^ + Fe^2+^ (2)

K^+^ + Fe^2+^ + Fe(CN)_6_^3–^ → KFeFe(CN)_6_ (3)

Formation of PB can be described by Reactions (2) and (3), see [6], whereby Fe(III) from Reaction (1) was further reduced to Fe(II), which then reacted with K^+^ and Fe(CN)_6_^3−^ to form PB. Immobilisation of Fe(II) ions on the CNT modified electrode might result from the interactions between Fe(II) ions and CNT surfaces with plenty of oxygen-containing functional groups [41,42,43]. The formation of PB deposits was evidenced by AFM, SEM and UV–Visible spectroscopy. The presence of CNT materials improved the formation of PB deposits.

### 2.3. Electrochemical and Electrocatalytic Properties of the GC/CNT/PB Modified Electrode

**Figure 4:**
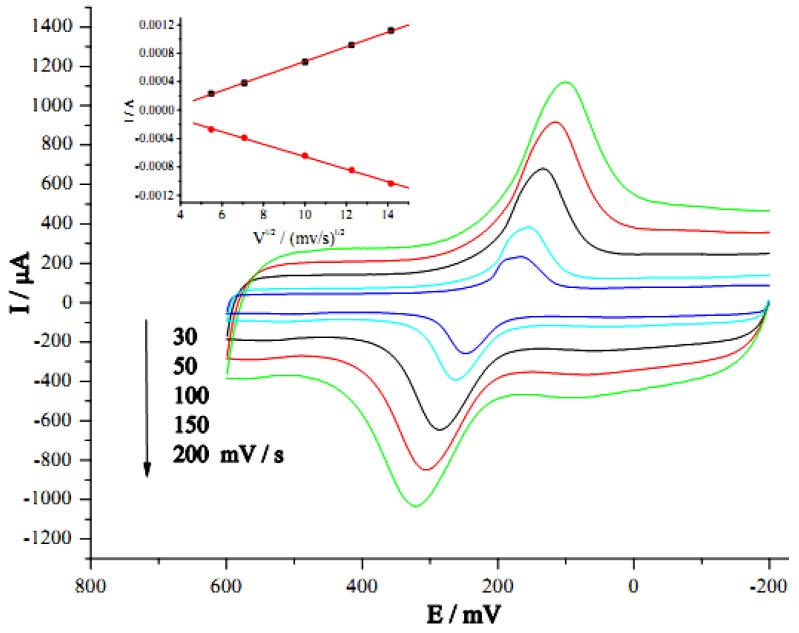
Cyclic voltammograms of GC/CNT/PB electrode in 0.1 M KCl at different scan rates. Inset: plot of peak current *vs*. square root of scan rate.

The electrochemical behavior of the CNT-modified electrode with PB deposits was examined by cyclic voltammetry in 0.1 M KCl at different scan rates. Figure 4 shows the effects of scan rate on the current response. The PB deposits showed quasi-reversible behavior with a peak current ratio of 1.1 (*I*_pc_/*I*_pa_) and a peak separation of 150 mV at a scan rate of 100 mV/s. Both reduction and oxidation peak current of PB deposits increased proportionally with the square root of the scan rate from 30 to 200 mV/s, with a correlation coefficient of approximately 0.999 (*N* = 5) for both processes. This suggested that the surface redox reactions were of diffusion-controlled behaviors [50]. These electrochemical characteristics demonstrated that the CNT/PB modified electrode showed good electrochemical activity of PB.

**Figure 5:**
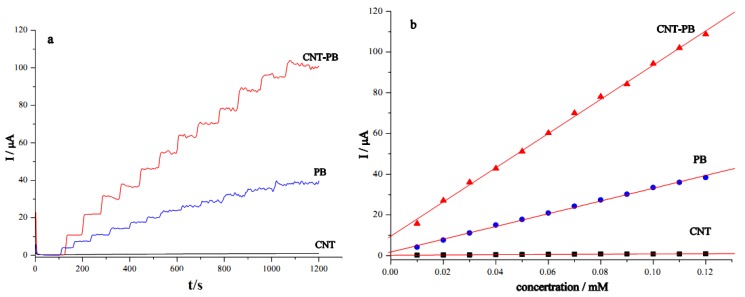
(**a**) Amperometric determination of H_2_O_2_ for GC electrodes modified with different materials: CNT-PB, PB, and CNT at −0.05 V (*vs.* Ag|AgCl) with successive addition of 10 μM H_2_O_2_ in phosphate buffer (pH 7.4). (**b**) Calibration plots for H_2_O_2_ determination at different modified GC electrodes.

H_2_O_2_ is a by-product of enzymatic reactions involving different oxidase enzymes. For an oxidase-based biosensor, detection of H_2_O_2_ at low potential is advantageous for both high sensitivity and high selectivity in the detection of analytes. PB offers effective electron transfer for H_2_O_2_ detection. The reduction of H_2_O_2_ at different modified electrodes (GC/CNT/PB, GC/PB, and GC/CNT) was examined. Figure 5a shows the amperometric response curves for the reduction of H_2_O_2_ examined at −0.05 V by successive addition of 10 μM H_2_O_2_ into the phosphate buffer (pH 7.4). Usually, the amperometric current response exhibited a stepwise increase upon addition of H_2_O_2_ and reached equilibrium within 2 s, for the electrodes examined in this study. The electrode modified with CNT alone did not favor the reduction of H_2_O_2_ at −0.05 V. In contrast, the electrode modified with PB only showed activity of H_2_O_2_ reduction. Interestingly, a much enhanced current response was observed for the GC/CNT/PB modified electrode, demonstrating the synergistic effects of PB and CNT. The GC/CNT/PB electrode responded more sensitively to H_2_O_2_, and showed a three-fold increase in response current compared with that for the GC/PB electrode. The GC/CNT/PB electrode can be applied for a practical determination of H_2_O_2_.

The corresponding calibration plots (current response *versus* hydrogen peroxide concentration) for the detection of H_2_O_2_ at different modified electrodes are shown in Figure 5b. The modified electrodes showed linear responses to increasing H_2_O_2_ concentration. The GC/CNT/PB modified electrode exhibited the best performance for electrocatalytic reduction of H_2_O_2_. The sensitivity of H_2_O_2_ reduction at the GC/CNT/PB modified electrode was found to be 13.0 μA mM^−1^ cm^−2^ for an electrode surface area of 0.0707 cm^2^, about 14-fold higher than that obtained at the CNT-modified electrode and nearly threefold larger than that at the PB-modified electrode. The GC/CNT/PB modified electrode showed much better sensitivity for the detection of H_2_O_2_ and a detection limit of 1 μM as compared to the electrodes modified with multi-layer composites of CNT/PB hybrid material obtained by layer-by-layer assembly with poly(diallyldimethylammmonium chloride) coatings reported elsewhere [51]. The layer-by-layer CNT/PB electrode system offered a sensitivity of 0.83 μA mM^−1^ cm^−2^ and a linear range from 1 to 10 μM [51]. On the other hand, the PB modified electrodes (GC/CNT/PB and GC/PB) offered wider linear range (10–120 μM) for the amperometric determination of H_2_O_2_. Fast response times of less than 0.2 s were observed for the three modified electrodes. Experimental results are summarized in Table 1.

**Table 1:** Electrode performance for H_2_O_2_ reduction at −0.05 V (*vs.* Ag|AgCl).

Electrode	GC/CNT	GC/PB	GC/CNT/PB
Sensitivity (AM^−1^cm^−2^)	0.82	4.7	13.0
Linear range (μM)	10–60	10–120	10–120
Detection limit (μM)	2	2	1
Response time (s)	<0.2	<0.2	<0.2

Prussian blue (PB), carbon nanotube (CNT).

### 2.4. Preparation and Performance Characteristics of the GC/CNT/PB/PPD-GOD Glucose Biosensor

Electropolymerized polyphenylenediamine (PPD) films have been utilized for the development of amperometric glucose biosensors offering good selectivity towards common interferences such as ascorbic acid and uric acid [52,53]. The preparation of CNT-based glucose biosensors with electropolymerized PPD film has been reported in detail elsewhere [54,55]. The PPD film obtained was usually of a thin film, enabling fast diffusion of the substrates and products through the polymer matrix. The resulting PPD modified electrodes usually showed rapid electrochemical responses to hydrogen peroxide and reached equilibrium within 2 s [56]. Electropolymerized PPD film was thus utilized for the co-immobilisation of GOD enzymes at the CNT/PB modified electrodes in this study.

**Figure 6:**
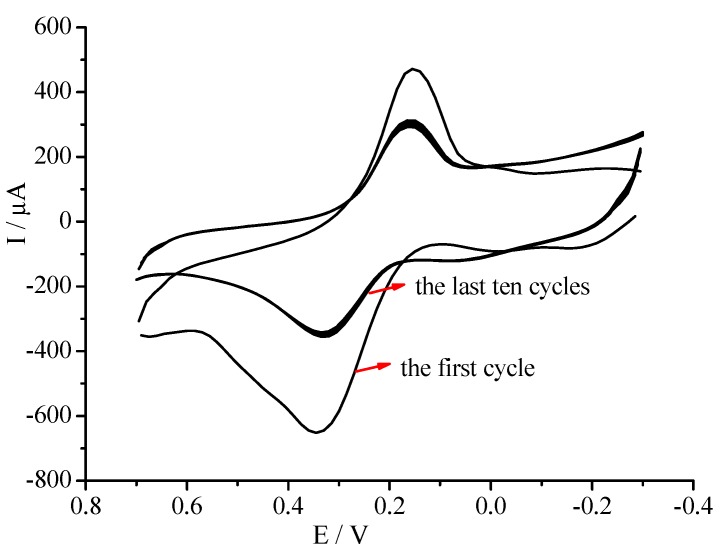
Cyclic voltammograms of GC/CNT/PB modified electrode in phosphate buffer (pH 7.4) containing 5 mM phenylenediamine and 10 mg/mL glucose oxidase (GOD) at a scan rate of 50 mV/s.

Glucose oxidase was immobilized on the GC/CNT/PB surface through electropolymerization of 5 mM phenylenediamine containing 10 mg/mL GOD between −0.30 and +0.70 V for 60 cycles in the present work. Cyclic voltammograms for co-immobilisation of PPD and GOD at the GC/CNT/PB electrode are shown in Figure 6. Oxidation of phenylenediamine was observed at potentials more positive than +0.60 V. The redox peaks at +0.15 and +0.35 V corresponded to the redox switching of the Prussian blue/Prussian white couple. After the first cycle, a sharp decrease in the oxidation current of phenylenediamine was observed, due to the presence of the nonconducting PPD film on the electrode surface. As the scanning number increased, the oxidation current reached a constant value, indicating the polymerization process was self-terminated [54,55]. Upon continuous voltammetric cycling, PPD film formed on the GC/CNT/PB modified surface. The peak current for PB deposits gradually decreased and reached constant values after 50 cycles. It indicated that the PB film was stable.

**Figure 7:**
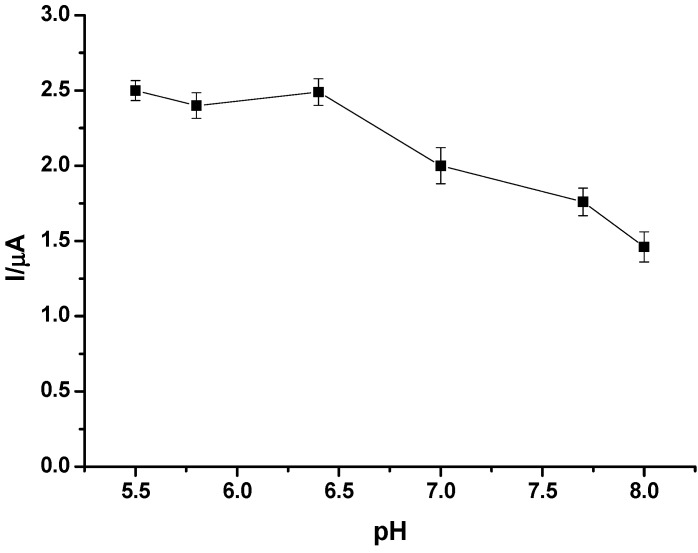
Effects of pH on the amperometric performance of GC/CNT/PB/PPD-GOD electrode to glucose at an applied potential of −0.05 V (*vs.* Ag|AgCl) in phosphate buffer (pH 7.4).

One of the disadvantages of many PB modified electrodes is their fast deterioration of sensitivity in neutral and alkaline pH [57,58,59]. Therefore, the pH of the solution medium is usually an important factor for effective performance of amperometric biosensors. The sensitivity of a biosensor is usually pH dependent and glucose detection is commonly conducted under physiological conditions. The influence of pH on the current response for glucose determination at the GC/CNT/PB/PPD-GOD modified electrode was investigated, and the experimental results are shown in Figure 7. The biosensor exhibited good bioactivity over a pH range from weakly acidic (pH 5.5) solution to slightly basic (pH 8.0) media. It suggested that the pH of the medium had little effect on the detection of glucose, and the PB deposits on the CNT-modified electrode were relatively stable. A phosphate buffer of pH 7.4, consistent with the physiological condition, was used as the supporting electrolyte in this work.

**Figure 8:**
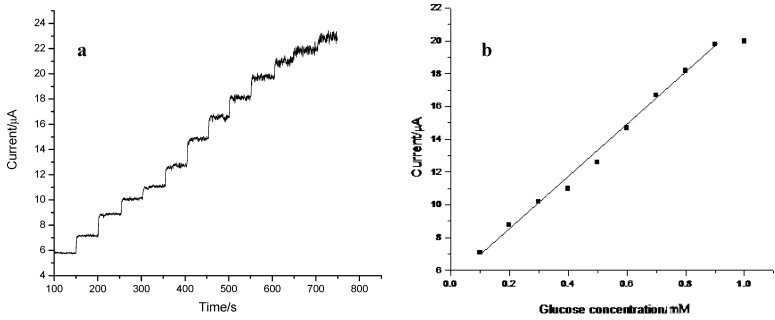
(**a**) Amperometric response of GC/CNT/PB/PPD-GOD electrode at −0.05 V (*vs.* Ag|AgCl) with successive addition of 0.1 mM glucose in phosphate buffer (pH 7.4); (**b**) Calibration plot for glucose determination at the GC/CNT/PB/PPD-GOD electrode.

Reduction of H_2_O_2_ at a GC/PB modified electrode usually occurred at a negative potential between 0.0 and −0.2 V *versus* Ag|AgCl, where biosensors exhibited the highest sensitivity and the lowest interference signal [60]. The amperometric response of the GC/CNT/PB/PPD-GOD electrode to glucose at a low detection potential of −0.05 V was examined, and the corresponding electrode response is shown in Figure 8a. The current response of the GC/CNT/PB/PDD-GOD electrode increased with increasing glucose concentration, while the GC/CNT/PB electrode without GOD enzyme did not respond to glucose. The corresponding calibration plot for glucose detection at the GC/CNT/PB/PDD-GOD electrode is shown in Figure 8b. The sensitivity of the glucose biosensor was 7.21 μA mM^−1^ (or 0.102 A M^−1^ cm^−2^) with a linear range from 0.1 to 0.9 mM and a detection limit of 10 μM at a signal-to-noise ratio of 3. Table 2 shows a comparison of the electrode response between the present biosensor with other glucose biosensors reported in the literature [45,61,62,63,64,65,66]. Our biosensor offered much higher sensitivity compared with other glucose biosensor systems utilizing PB [63,64,66]. The results demonstrated that the biosensor based on the spontaneous deposition of PB on the GC/CNT surface offered sensitive detection of glucose and exhibited excellent electrocatalytic behavior toward glucose.

**Table 2:** Performance of different electrode systems for glucose determination.

Electrode	Linear range (μM)	Detection limit (μM)	Sensitivity (mA M^−1^ cm^−2^)
GC/CNT/PB/PPD-GOD [this work]	100–900	10	102
GC/Pt-CNT-GOD-Nafion [61]	160–11500	55	------
GC/CNT/CS/Cu [62]	50–1200	20	-----
GC/Au-MWNT/GOD/Nafion [63]	50–2200	20	5.66
Pt/PB/GA-GOD [64]	5–1100	5	43
GC/PB-CS/GOD [65]	2–400	0.397	-----
GC/MWNTs/PB/PDAB/GOD [45]	10–2500	5	-----
GC/CS/MWNTs/PB/GOD [66]	4–2000	2.5	7.84

Glucose oxidase (GOD), electropolymerized polyphenylenediamine (PPD).

**Figure 9:**
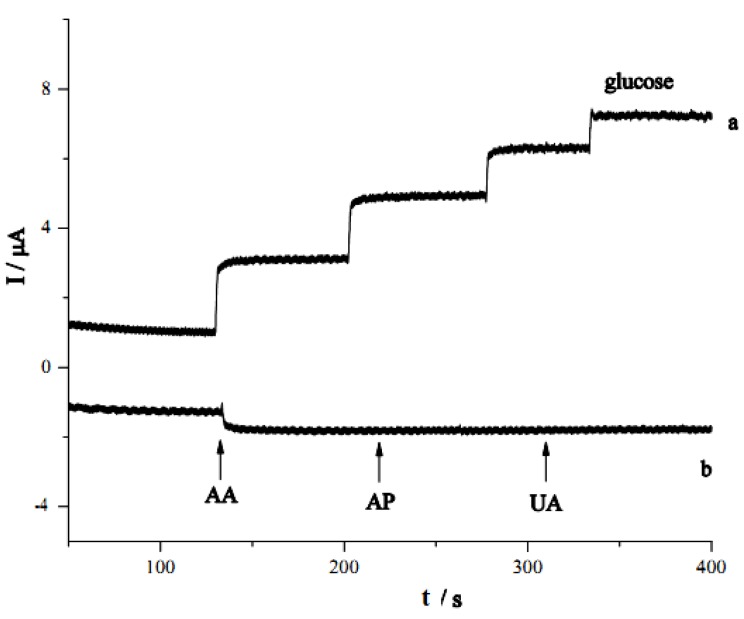
(**a**) Amperometric response of GC/CNT/PB/PPD-GOD electrode with successive addition of glucose; (**b**) Amperometric response of GC/CNT/PB/PPD-GOD electrode with the addition of 0.1 mM ascorbic acid (AA), 0.1 mM acetaminophen (AP) and 0.02 mM uric acid (UA). Applied potential: at −0.05 V (*vs.* Ag|AgCl); Supporting electrolyte: phosphate buffer (pH 7.4).

Figure 9 shows the current response of the GC/CNT/PB/PPD-GOD electrode to glucose and common interferences at an applied potential of −0.05 V. Upon addition of 0.1 mM glucose, a current of 1.95 μA was observed. A smaller response current of 0.51 μA was realized when 0.1 mM ascorbic acid (AA) was added. Continuous addition of 0.1 mM acetaminophen (AP) and 0.02 mM uric acid (UA) to the solution caused no obvious change (*i.e.*, <2%) in the response current. This suggested that AA, AP and UA did not interfere with the glucose detection at the GC/CNT/PB/PPD-GOD electrode to a great extent at an applied potential of −0.05 V.

The stability of the glucose biosensor was also examined by amperometric detection of 0.1 mM glucose solution. The biosensor was stored in phosphate buffer in a refrigerator at 4 °C and the electrode response was examined every two days. The current response showed no significant decrease within three weeks. The current decreased to 70% of the original value after two months. PB deposits obtained by spontaneous deposition offered high stability.

## 3. Experimental Section

### 3.1. Chemicals and Instruments

Multi-walled carbon nanotubes were purchased from Nanotech Port Co. Ltd. (Shenzhen, China) and were purified with 10 M HNO_3_ for ten hours before use. Glucose oxidase (GOD) (Type X-S, from *Aspergillus niger*), β-D(+)-glucose, 1,2-phenylenediamine, ascorbic acid, acetaminophen and uric acid were obtained from Sigma-Aldrich. H_2_O_2_ was obtained from BDH. Potassium hexacyanoferrate(III) was purchased from International Laboratory. All other reagents were of reagent grade and were used without further purification. Phosphate buffer (pH 7.4) consisting of 0.2 M KH_2_PO_4_, 0.2 M NaOH and 0.1 M KCl was used as the supporting electrolyte. All solutions were prepared using deionised water.

Surface characterization of the PB and CNT/PB modified electrodes was performed using a scanning electron microscope (LEO, Electron Microscopy Inc., Cambridge, UK) and an atomic force microscope (SPA-300 HA, Seiko Instruments Inc., Chiba, Japan). Ultraviolet–Visible absorption spectra were recorded using a Cary 100-Scan UV–Visible spectrophotometer (Varian Inc., Walnut Creek, CA, USA).

All electrochemical experiments were carried out by a BAS100BW electrochemical workstation (Bioanalytical Systems, Inc., West Lafayette, IN, USA). A traditional three-electrode system consisting of a glassy carbon working electrode, a platinum wire counter electrode and a Ag|AgCl reference electrode was used. All potentials were quoted *versus* the Ag|AgCl reference electrode.

### 3.2. Preparation of CNT/PB Modified Electrode

Glassy carbon electrodes with 3-mm diameter (Bioanalytical Systems, Inc., West Lafayette, IN, USA) were carefully polished with alumina slurry on a microcloth (Buehler, Lake Bluff, IL, USA), followed by ultrasonication in ethanol and deionized water. The electrodes were allowed to dry in air. Indium tin oxide (ITO) glass and silica glass were cleaned in an ultrasonic bath with ethanol and deionized water for 10 min before use.

In our work, 2 mg multi-walled CNTs with diameter of 10–30 nm was dispersed in deionized water by ultrasonic agitation to obtain a black suspension. Next, 10 µL of the suspension was applied to the GC electrode or ITO glass substrate, and was allowed to dry in air.

Deposition of PB on different electrode substrates was carried out by incubating the respective electrode in 0.1 M KCl (pH 1.6) containing 1 mM acidic K_3_Fe(CN)_6_ overnight. The electrode was rinsed with deionised water and was allowed to dry in air.

### 3.3. Spectroscopic Characterization of Electrode Substrates

Glassy carbon disks of 6 mm diameter were obtained by wire-cutting a GC rod (Atomergic Chemetals Corp., Farmingdale, NY, USA) into 2-mm thick pieces. The disk was then mounted onto a Teflon sheath. The disk electrode was polished and modified with CNT materials. The electrode surface was then modified with PB or CNT/PB deposits. The modified surface was then removed from the Teflon sheath and subjected to SEM and AFM measurements.

A drop of CNT suspension was dispersed onto a quartz glass slide and was allowed to dry in air. The CNT-coated glass slide was immersed in PB mixture for the preparation of the CNT/PB substrate. UV–Visible absorption spectra were then recorded with a UV–Visible Spectrophotometer.

### 3.4. Preparation of CNT/PB Modified Glucose Biosensor

Glucose biosensors were prepared by immersing the GC/CNT/PB electrode in phosphate buffer (pH 7.4) containing 5 mM phenylenediamine and 10 mg/mL glucose oxidase (GOD). Cyclic voltammetry was carried out by scanning the potential between −0.30 and +0.70 V at a scan rate of 50 mV/s for 60 cycles, as described previously [54,55]. The resulting GC/CNT/PB/PPD-GOD glucose biosensor was rinsed with deionized water thoroughly and was stored in a phosphate buffer (pH 7.4) before use. All measurements were performed at room temperature.

### 3.5. Amperometric Determination

The steady state amperometric response to H_2_O_2_ and glucose was voltammetrically measured in a gently stirred 0.1 M phosphate buffer containing 0.1 M KCl (pH 7.4) at the desired potentials. The current response was recorded on successive addition of the substrates.

## 4. Conclusions

A simple method for the spontaneous deposition of Prussian blue particles on a carbon nanotube modified electrode from a solution containing only the Fe(CN)_6_^3−^ species was demonstrated. The dissociation of Fe(CN)_6_^3−^ gave Fe(III) species which were further reduced to Fe(II) ions. Fe(II) ions immobilised on the CNT modified electrode surfaces with plenty of oxygen-containing functional groups and then reacted with K^+^ and Fe(CN)_6_^3−^ to form PB.

The resulting GC/CNT/PB electrode offered more than twofold increase in sensitivity for the reduction of H_2_O_2_ compared to electrodes modified with PB or CNT alone. An amperometric glucose biosensor was obtained by co-immobilization of glucose oxidase and PPD at the surface of the GC/CNT/PB modified electrode. The biosensor offered a linear response to glucose concentration from 0.1 to 0.9 mM with a high sensitivity of 0.102 A M^−1^ cm^−2^ and short response time at a negative operation potential of −0.05 V (*vs.* Ag|AgCl). The detection limit was estimated to be 0.01 mM. Common interference from chemicals such as ascorbic acid, acetaminophen and uric acid did not cause significant interference in this case for the detection of glucose.
